# Supplementary material for: Combined detection of serum IL-6 and CEA contributes to the diagnosis of lung adenocarcinoma in situ
Source: PeerJ. 2024 Mar 22;12:e17141. doi: 10.7717/peerj.17141 (PMC10962332; doi:10.7717/peerj.17141)
Supplement: Supplemental Information 1 — Boxplot, scatter of serum IL-6 (A), and the ROC curve of serum IL-6 (B). [file peerj-12-17141-s001.doc]

SUPPLEMENTARY MATERIALS


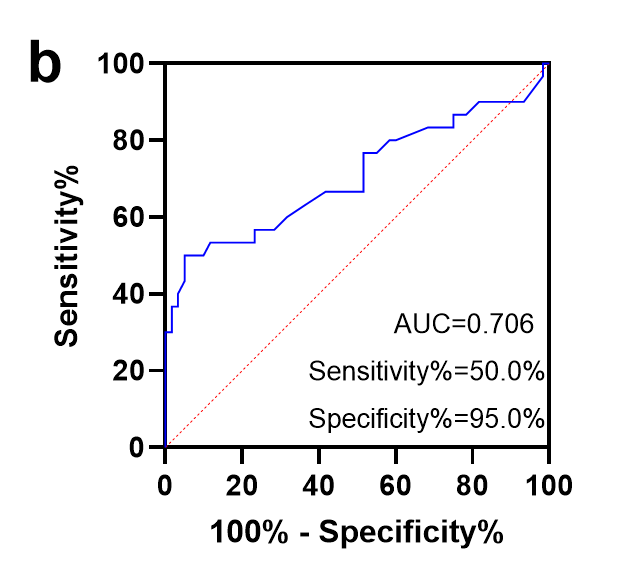

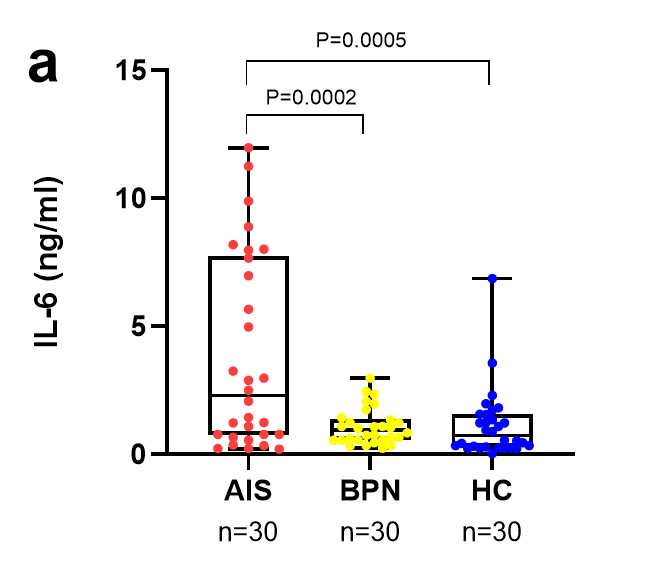

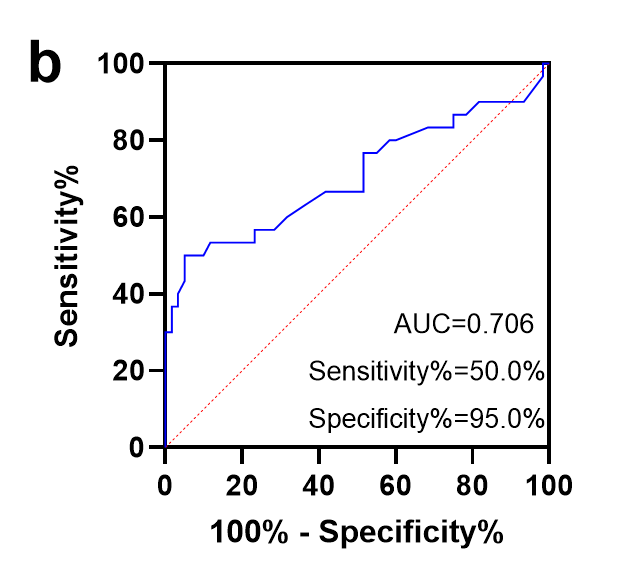

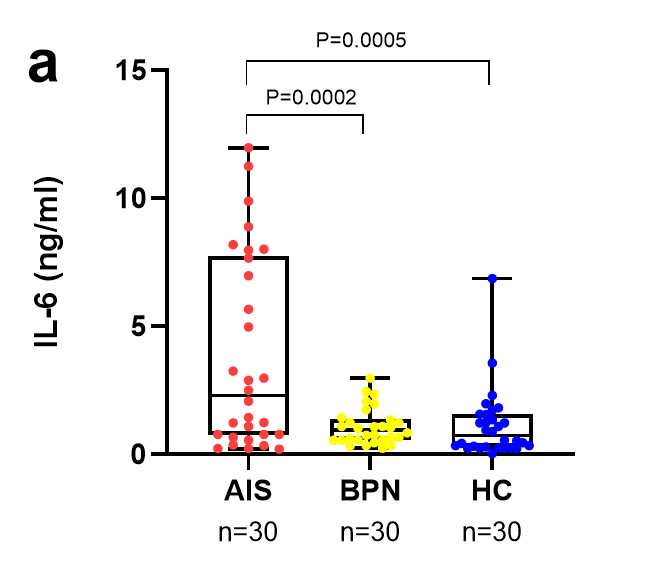


**Supplementary Fig.1.** Performance of serum IL-6 for AIS patients in a small sample cohort. Boxplot, scatter of serum IL-6 (a), and the ROC curve of serum IL-6 (b).
